# Ozone-induced changes in the serum metabolome: Role of the microbiome

**DOI:** 10.1371/journal.pone.0221633

**Published:** 2019-08-27

**Authors:** Youngji Cho, Ross S. Osgood, Lauren N. Bell, Edward D. Karoly, Stephanie A. Shore

**Affiliations:** 1 Department of Environmental Health, Harvard T.H. Chan School of Public Health, Boston, Massachusetts, United States of America; 2 Metabolon Inc., Durham, North Carolina, United States of America; West Virginia University School of Medicine, UNITED STATES

## Abstract

Ozone is an asthma trigger. In mice, the gut microbiome contributes to ozone-induced airway hyperresponsiveness, a defining feature of asthma, but the mechanistic basis for the role of the gut microbiome has not been established. Gut bacteria can affect the function of distal organs by generating metabolites that enter the blood and circulate systemically. We hypothesized that global metabolomic profiling of serum collected from ozone exposed mice could be used to identify metabolites contributing to the role of the microbiome in ozone-induced airway hyperresponsiveness. Mice were treated for two weeks with a cocktail of antibiotics (ampicillin, neomycin, metronidazole, and vancomycin) in the drinking water or with control water and then exposed to air or ozone (2 ppm for 3 hours). Twenty four hours later, blood was harvested and serum analyzed via liquid-chromatography or gas-chromatography coupled to mass spectrometry. Antibiotic treatment significantly affected 228 of the 562 biochemicals identified, including reductions in the known bacterially-derived metabolites, equol, indole propionate, 3-indoxyl sulfate, and 3-(4-hydroxyphenyl)propionate, confirming the efficacy of the antibiotic treatment. Ozone exposure caused significant changes in 334 metabolites. Importantly, ozone-induced changes in many of these metabolites were different in control and antibiotic-treated mice. For example, most medium and long chain fatty acids declined by 20–50% with ozone exposure in antibiotic-treated but not control mice. Most taurine-conjugated bile acids increased with ozone exposure in antibiotic-treated but not control mice. Ozone also caused marked (9-fold and 5-fold) increases in the polyamines, spermine and spermidine, respectively, in control but not antibiotic-treated mice. Each of these metabolites has the capacity to alter airway responsiveness and may account for the role of the microbiome in pulmonary responses to ozone.

## Introduction

Ozone (O_3_) is an air pollutant produced by interactions between sunlight and automobile exhaust. O_3_ causes injury to the airway epithelium leading to an inflammatory response that includes production of numerous cytokines and chemokine and recruitment of neutrophils to the lungs [1, 2]. O_3_ also causes respiratory symptoms [3] and reductions in lung function [4, 5] and is a particular problem in those with underlying cardiorespiratory disease, including asthma [3, 6–9]. Indeed, O_3_ causes airway hyperresponsiveness (AHR) [10], a canonical feature of asthma. The number of asthma hospital admissions and emergency room visits is elevated following days when ambient O_3_ concentrations are high [11–13].

In male C57BL/6 mice, the gut microbiome contributes to the ability of O_3_ to induce AHR and recruit neutrophils to the lungs [1]. When conventionally raised mice are treated, via their drinking water, with a cocktail of antibiotics prior to acute O_3_ exposure, O_3_-induced AHR and O_3_-induced increases in BAL neutrophils are reduced. O_3_-induced AHR and O_3_-induced increases in BAL neutrophils are also reduced in germ-free (GF) versus conventionally housed (CONV) mice. The gut microbiome is altered in obesity and O_3_-induced AHR is greater in obese than lean mice. Moreover, compared to transplant with gut microbiota from lean mice, transplant of GF mice with gut microbiota from obese mice augments O_3_-induced AHR and inflammation [14]. The observations that oral administration of vancomycin reduces O_3_-induced AHR [1] but does not affect the lung microbiome [15] indicates that it is likely the gut rather than the lung microbiome that affects pulmonary responses to O_3_. Nevertheless, the mechanistic basis for the role of the microbiome has not been established.

Gut bacteria can affect the function of distal organs by generating metabolites from dietary factors and by modifying host-derived metabolites [16]. Such metabolites can enter the blood and circulate systemically. Indeed, metabolomic profiling of blood from GF versus CONV mice or from antibiotic-treated versus control mice [17–19] indicates bacteria-related differences in some of the metabolites present. These metabolites can influence host physiology. For example, trimethylamine N-oxide (TMAO), which has been linked to cardiovascular disease, is produced by bacterial metabolism of dietary choline to TMA which is then oxidized in the liver [20, 21]. The gut microbiome is altered in patients with autism spectrum disorder and gut microbiota from such patients elicit a similar disorder when transplanted into GF mice, likely as a result of decreased bacterial production of 5-aminovaleric acid, a weak GABA receptor agonist [22]. Bacterial metabolism of dietary fiber leading to production of short chain fatty acids (SCFAs) and bacterial arginine metabolism leading to production of polyamines are both associated with altered immune function [16].

In rats, acute exposure to O_3_ causes profound changes in the serum metabolome including increases in sugars, free fatty acids, branched chain amino acids (BCAAs), and urea, indicating impaired glycemic control, lipolysis, and proteolysis [23]. Importantly, these systemic effects of O_3_ appear to contribute to O_3_-induced injury and inflammation within the lungs [24]. We have established that in mice, O_3_ also impacts the lung metabolome [25], leading to changes in glutathione metabolism, phospholipid metabolism, and changes in the metabolism of BCAAs.

We hypothesized that global metabolomic profiling of serum collected from O_3_-exposed mice could be used to identify metabolites contributing to the role of the microbiome in pulmonary responses to O_3_ [1]. To that end, mice were treated with a cocktail of antibiotics in the drinking water or with regular drinking water for 14 days prior to exposure. Mice were exposed to room air or to O_3_ (2 ppm) for 3 hours. The dose of O_3_ was chosen because it causes microbiome-dependent AHR in mice [1]. Because of differences in dosimetry between humans and mice, the inhaled dose of O_3_ produced by the exposure regimen we used is approximately equivalent to the inhaled dose produced in humans chamber studies in which O_3_-induced AHR was evaluated using O_3_ concentrations of 400 ppb [26]. The latter concentration is still greatly in excess of the US Environmental Protection Agency standard but approaches peak concentrations sometimes observed in several highly populated cities [27, 28]. O_3_-induced AHR peaks approximately 24 h after cessation of exposure [2]. Consequently, 24 hours after exposure, blood was harvested, serum was prepared and frozen until being analyzed via liquid-chromatography or gas-chromatography coupled to mass spectrometry for metabolites. Our data indicated marked effects of both O_3_ exposure and antibiotics on the serum metabolome. Importantly, our data also indicated that O_3_-induced changes in serum lipids, including long chain fatty acids and bile acids, as well as O_3_-induced changes in polyamines are different in control and antibiotic-treated mice. Each of these metabolites has the capacity to alter airway responsiveness and neutrophil recruitment and thus affect the response to O_3_ and may contribute to the role of the microbiome in pulmonary responses to O_3_

## Materials and methods

### Mice

Male C57BL/6 mice aged 6–7 weeks were purchased from Taconic Farms (New York) and held in the Harvard T.H. Chan School of Public Health vivarium for one week prior to the initiation of antibiotic (or regular water) treatment. The study was approved by the Harvard Medical Area Standing Committee on Animals (protocol 03753). We chose to examine C67BL/6 male mice because we have established the gut microbiome is required for O_3_-induced AHR in this sex and strain [1]. Mice were housed under a 12 hour lights on/off cycle and were fed standard mouse chow.

### Protocol

Mice (n = 8 per group) were treated with a cocktail of antibiotics in the drinking water for 2 weeks, as previously described [1]. We and others have reported that this cocktail causes a marked depletion of most gut microbial taxa [1, 29]. This cocktail included: ampicillin 1g/L, metronidazole 1g/L, neomycin 1g/L, vancomycin 0.5 g/L. Sucralose (8 g/L) was added to this antibiotic-treated drinking water to make it more palatable. Note that control mice were given regular drinking water with the same concentration of sucralose. In a separate cohort of male C57BL/6 mice treated in an identical fashion, body weight before and after 2 weeks of drinking the sucralose-treated control water averaged 26.3 ± 0.7 and 27.4 ± 0.6 g, respectively. Similarly, in the antibiotic-treated mice, body weight averaged 28.0 ± 0.7 and 29.0 ± 0.7 g before and after 2 weeks of antibiotic treatment, respectively. The increases in body weight in the antibiotic and control treated mice were not different and indicate no loss in body weight as would be evident if the mice reduced their water consumption. Mice were then exposed to O_3_ (2 ppm for 3 h) or to room air as previously described [1]. Since our goal was to identify bacterial metabolites that could be mediating the observed microbiome-dependent effects on responses to O_3_, we chose the same exposure regimen as we used when we initially reported those microbiome-dependent effects [1]. Harvesting of tissues was always done at the same time of the day (between 11am and 1 pm on the day after O_3_ exposure). Approximately 8 mice were euthanized on a given day. The order of harvest of the mice on a given day was randomized across groups.

### Ozone exposure

Mice were exposed to O_3_ (2 ppm for 3 hours) or to room air. During O_3_ exposure, mice were placed in individual wire mesh cages inside a 145 L stainless steel chamber with a plexiglass door without food or water. Air-exposed mice were placed in a separate but identical chamber. Immediately after exposure, mice were returned to their home cages and provided with food and control or antibiotic-containing water ad libitum. O_3_ was generated by passing oxygen through ultraviolet (UV) light. This gas was mixed with room air in the chamber. A sample of the chamber atmosphere was continuously drawn through a sampling port, the O_3_ concentration within the chamber monitored by a UV photometric O_3_ analyzer (model 49; Thermo Electron Instruments, Hopkinton, MA).

### Tissue harvest and processing

Twenty four hours after exposure, mice were euthanized with an overdose of sodium pentobarbital. Blood was obtained by cardiac puncture for the preparation of serum. Serum was stored at -80°C until shipped on dry ice to Metabolon Inc. (Durham, NC). Upon receipt, the serum was again frozen at -80°C until analysis.

### Metabolomics

#### Sample preparation

Samples were shipped to Metabolon for processing and prepared for metabolomics as previously described [30]. Briefly, an equivalent amount serum (100μl) on a per sample basis was prepared using the automated MicroLab STAR^®^ system from Hamilton Company. For QC purposes, a recovery standard was added prior to the first step in the extraction process. To remove protein, dissociate small molecules bound to protein or trapped in the precipitated protein matrix, and to recover chemically diverse metabolites, proteins were first precipitated with methanol under vigorous shaking for 2 min (Glen Mills GenoGrinder 2000) followed by centrifugation. The resulting extract was divided into five fractions as follows. One fraction was used for analysis by UPLC-MS/MS with positive ion mode electrospray ionization. Another fraction was used for analysis by UPLC-MS/MS with negative ion mode electrospray ionization. The third and fourth fractions were used for LC polar platform, and for analysis by GC-MS. The final fraction was reserved as a backup. Samples were placed briefly on a TurboVap^®^ (Zymark) to remove the organic solvent. For LC, the samples were stored overnight under nitrogen before preparation for analysis. For GC, each sample was dried under vacuum overnight before preparation for analysis.

#### Ultrahigh performance liquid chromatography-Tandem mass spectroscopy (UPLC-MS/MS)

The LC/MS portion of the platform was based on a Waters ACQUITY ultra-performance liquid chromatography (UPLC) and a Thermo Scientific Q-Exactive high resolution/accurate mass spectrometer interfaced with a heated electrospray ionization (HESI-II) source and Orbitrap mass analyzer operated at 35,000 mass resolution. Each sample extract was first dried, then reconstituted in acidic or basic LC-compatible solvents. To ensure injection and chromatographic consistency, these solvents each contained 8 or more injection standards at fixed concentrations. One aliquot was analyzed using acidic positive ion optimized conditions and the other using basic negative ion optimized conditions in two independent injections using separate dedicated columns (Waters UPLC BEH C18-2.1x100 mm, 1.7 μm). Extracts reconstituted in acidic conditions were gradient eluted from a C18 column using water and methanol containing 0.1% formic acid. The basic extracts were similarly eluted from C18 using methanol and water, however with 6.5mM Ammonium Bicarbonate. The third aliquot was analyzed via negative ionization following elution from a HILIC column (Waters UPLC BEH Amide 2.1x150 mm, 1.7 μm) using a gradient consisting of water and acetonitrile with 10mM Ammonium Formate. The MS analysis alternated between MS and data-dependent MS2 scans using dynamic exclusion, and the scan range was from 80–1000 m/z.

#### Gas Chromatography-Mass spectroscopy (GC-MS)

For GC-MS analysis, samples were dried under vacuum for at least 18 h prior to derivatization under dried nitrogen using bistrimethyl-silyltrifluoroacetamide. Derivatized samples were separated on a 5% diphenyl / 95% dimethyl polysiloxane fused silica column (20 m x 0.18 mm ID; 0.18 um film thickness) with helium as carrier gas and a temperature ramp from 60° to 340°C over a 17.5 min period. Samples were analyzed on a Thermo-Finnigan Trace DSQ fast-scanning single-quadrupole mass spectrometer using electron impact ionization (EI) and operated at unit mass resolving power. The scan range was from 50–750 m/z.

#### Metabolomics data extraction and compound identification

Raw data were extracted, peak-identified and QC processed using Metabolon’s proprietary hardware and software. Compounds were identified by comparison to library entries of purified standards or recurrent unknown entities. This library is based on authenticated standards and contains the retention time/index (RI), mass to charge ratio (*m/z)*, and chromatographic data (including MS/MS spectral data) on all molecules present in the library. Biochemical identification was based on three criteria: retention index within a narrow RI window of the proposed identification, accurate mass match to the library +/- 0.005 amu, and the MS/MS forward and reverse scores between the experimental data and authentic standards. The MS/MS scores are based on a comparison of the ions present in the experimental spectrum to the ions present in the library spectrum. The Metabolon library contains more than 3300 commercially available purified standard compounds. Peaks were quantified using area-under-the-curve. This raw data is available at the NIH Common Fund’s Metabolomics Data Repository and Coordinating Center website, the Metabolomics Workbench, http://www.metabolomicsworkbench.org, Project ID PR000764.

#### RNA extraction and real time PCR

Livers were excised from another cohort of male mice treated and exposed in an identical manner [1]. Livers were snap-frozen in liquid nitrogen and stored in -80°C, then thawed over ice for subsequent preparation of RNA [31]. We used a small volume spectrophotometer (Nanodrop, Thermo Scientific) to assess RNA concentration and purity. A commercial kit (SuperScript III for qRT-PCR, Invitrogen) was used to convert RNA into cDNA. Liver *Cyp7a1*, *Cyp7b1*, *Cyp8b1*, *Baat*, and *Slc10a1* mRNA abundances were quantified using real time PCR (7300 Real-Time PCR Systems, Applied Biosystems) with SYBR-green detection and normalized to *36B4* ribosomal RNA (*Rplp0)*. Primers for these genes were as follows: *Rplp0*: Forward: GCTCCAAGCAGATGCAGCA, Reverse: CCGGATGTGAGGCAGCAG. *Cyp7a1*: Forward: GGCATCTCAAGCAAACACCA, Reverse: GCTTTCATTGCTTCAGGGCT. *Cyp8b1*: Forward: CCCATAAGACGCCATCCCTC, Reverse: AAGTGTGGGTGAGCCATCAG. *Baat*: Forward: GGTTGCTGTAAAACTACTGTTTTGG, Reverse: TGTGCACAGGCTCATCAACA. *Slc10a1*: Forward: ACCCTACGTCCTCAAGGCAG, Reverse: AGCCAGTAAGTGTGGTGTCA. The ΔΔCt method was used to assess changes in the mRNA abundance.

#### Statistics

To assess the significance of differences in mRNA abundances, factorial ANOVA with treatment and exposure as main effects was used with LSD Fisher post-hoc analysis (Statistica Software, Tulsa, OK). For metabolomics data analysis, for each metabolite, raw peak area counts were rescaled to set the median across all samples to 1. A two way ANOVA using the factors, treatment and exposure, was then performed on log transformed data. Follow-up pairwise contrasts were performed using F-tests. Storey’s q-values were calculated to estimate the proportion of false positives. S1 Table contains these p and q values for each metabolite along with fold change values. Principal component analysis (PCA) was generated using ArrayStudio.

To determine whether there were metabolic pathways that were enriched for significantly affected metabolites, we calculated an enrichment factor (EF) as follows:

$$EF=\frac{\#\;\text{of}\;\text{significantly}\;\text{affected}\;\text{metabolites}\;\text{in}\;\text{pathway}/\text{total}\;\#\;\text{of}\;\text{metabolites}\;\text{in}\;\text{pathway}}{\text{total}\;\#\;\text{of}\;\text{significantly}\;\text{affected}\;\text{metabolites}/\text{total}\;\#\;\text{of}\;\text{detected}\;\text{metabolites}}$$

Chi-square tests were used to assess the significance of superpathway or pathway enrichment.

## Results

**Fig 1:**
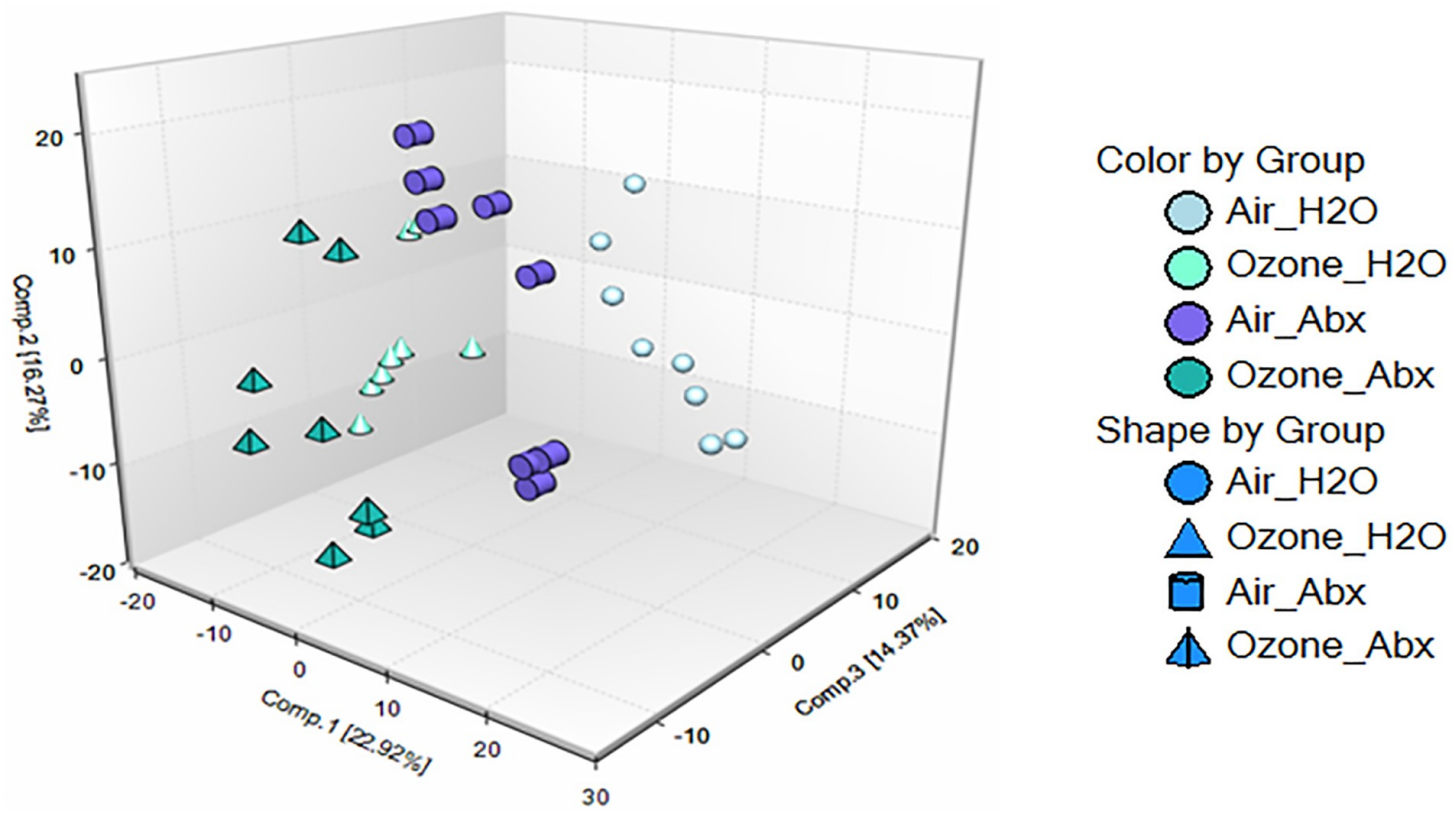
Principal component analysis. Serum metabolites from male C57BL/6 mice treated with regular drinking water or with a cocktail of antibiotics were analyzed. Treatment continued for 2 weeks at which time mice were exposed to air or ozone (2 ppm for 3 h) and studied 24 h after exposure. n = 8 per group.

Antibiotic treatment and O_3_ exposure each had profound effects on the metabolome of serum. Among the 562 named biochemicals identified in serum, two-way ANOVA identified 228 that were significantly (p<0.05 and q<0.10) affected by antibiotic treatment, 334 that were affected by O_3_ exposure, and 185 for which there was an interaction between antibiotic treatment and O_3_. Differences between individual experimental groups are shown in Table 1. The particular serum metabolites that were significantly affected by antibiotic treatment or by O_3_ exposure are highlighted in red (increased) and green (decreased) in S1 Table. Boxplots showing differences across the four groups are shown in S2 Table. The metabolic superpathways and pathways to which these metabolites belong are also indicated in this table. Principal component analysis (PCA) also indicated a clear separation in the serum metabolomes of the antibiotic- versus water-treated mice and the O_3_- versus air-exposed mice (Fig 1). Below we first describe differences in the serum metabolomes of antibiotic- versus water-treated mice that had been exposed only to room air. The purpose of this analysis is to allow us to determine which metabolites in the serum are affected by the microbiome. This analysis also serves as a positive control indicating the efficacy of the antibiotic treatment. We then describe the impact of O_3_ exposure on the serum metabolome of the water-treated mice. Finally, we present data indicating which metabolites were differentially impacted by O_3_ in antibiotic- versus water-treated (control) mice.

**Table 1:** Total number of serum biochemicals that were significantly affected by ozone and antibiotic treatment.

*ANOVA* *Contrasts*	Antibiotics Water		Ozone Room air	
	Air	Ozone	Water	Antibiotics
Total biochemicals p≤0.05and q≤0.10	244	144	257	291
Biochemicals (↑/↓)	146/98	46/98	92/165	54/237

### Effect of antibiotics in air-exposed mice

**Fig 2:**
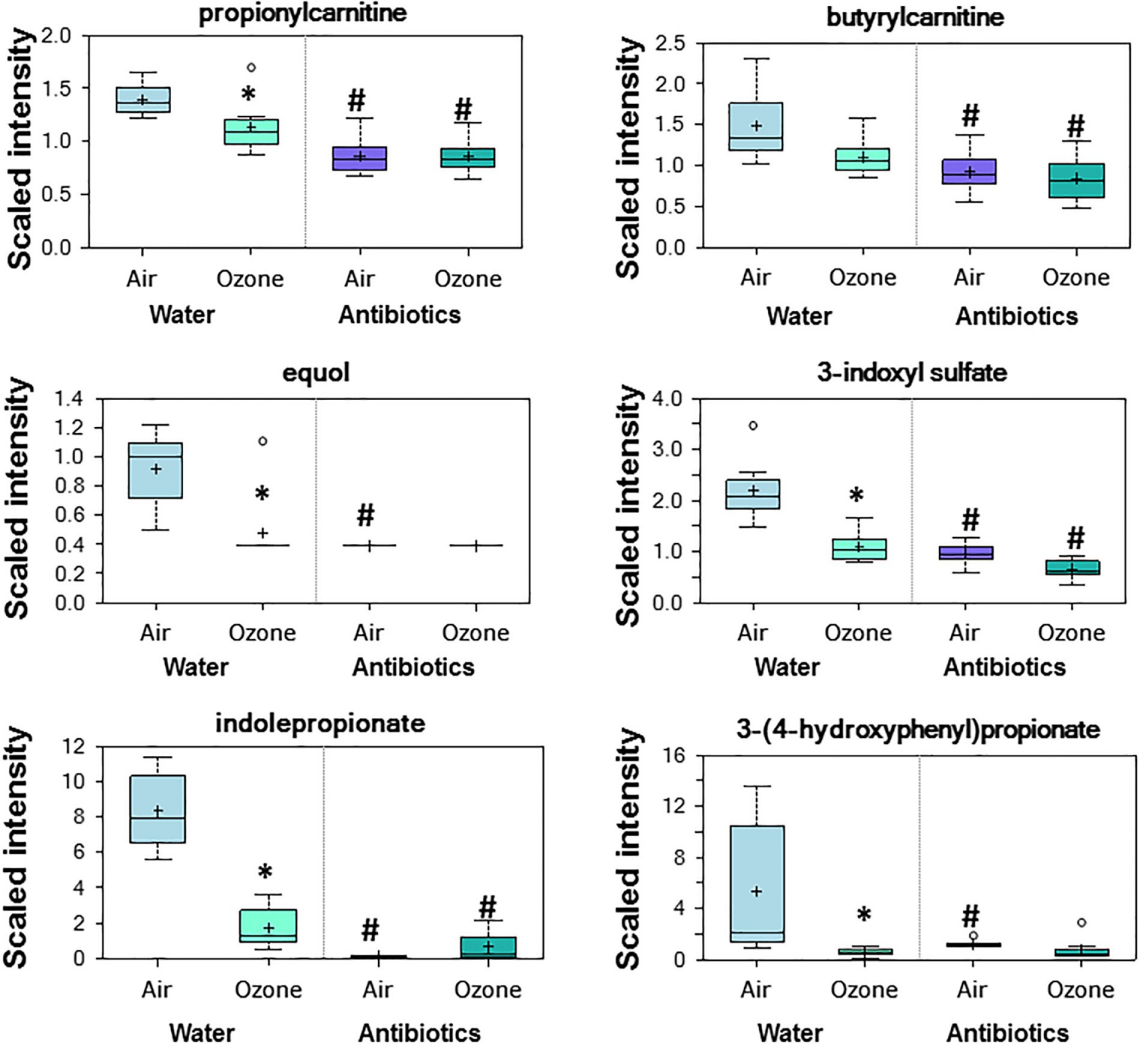
Bacterial-derived metabolites in serum of mice treated with water or antibiotics prior to air or ozone. For each metabolite, peaks were quantified using area-under-the-curve. Then, the median area-under-the-curve for that metabolite across all mice in all groups was calculated. Then, a scaled intensity for each metabolite was calculated by dividing the raw area counts for each mouse by this median so that the median across all mice in all groups was now equal to 1. This scaled intensity is presented on the y axis. For each group, the + indicates the mean value and the line in the center of bar indicates the median. The upper and lower limits of the bar are the upper and lower quartile and the top and bottom of the error bars are the maximum and minimum of the distribution. Extreme data points are indicated by symbols outside of the maximum and minimum of the distribution. # p<0.05 and q<0.10 versus water-treated mice with the same exposure; * p<0.05 and q<0.10 versus air exposed mice with same treatment. n = 8 per group.

In air-exposed mice, antibiotic treatment caused significant changes in serum biochemicals within multiple metabolic pathways (S1 Table—see metabolites highlighted in green (decreased) or red (increased) in column Q). These changes included reductions in many biochemicals whose generation is known to require bacterial enzymes, confirming the efficacy of the antibiotic treatment. For example, propionylcarnitine, butyrlcarnitine, equol, indole propionate, 3-indoxyl sulfate, and 3-(4-hydroxyphenyl)propionate were each significantly lower in serum of antibiotic-treated versus water-treated mice (Fig 2). Propionylcarnitine and butyrlcarnitine are the carnitine derivatives of the SCFAs, propionate and butyrate, which are produced by bacterial metabolism of dietary fiber [32], though they can also be produced through mammalian enzyme pathways. Gut bacteria generate equol from dietary daidzein, a component of mouse chow [33]. Indoles are produced by gut bacterial metabolism of dietary tryptophan [34] and 3-(4-hydroxyphenyl)propionate is produced by gut bacterial metabolism of dietary flavonoids [35]. Other known microbial or microbial-mammalian metabolites [18, 36–38], especially metabolites of aromatic amino acids, benzoate, and various food components were also substantially and significantly decreased in serum of antibiotic- versus water-treated mice exposed to air (see S1 Table—metabolites highlighted in green or red in column Q, rows 52–98 (aromatic amino acids), rows 508–520 (benzoate), and rows 521–551 (food components)).

Treatment with antibiotics also caused a significant, 18% reduction in serum glucose (S1 Table, column Q, row 193), consistent with previous reports describing serum of GF versus CONV mice [39]. In addition, antibiotics increased serum levels of several medium chain fatty acids, and polyunsaturated fatty acids (n3 and n6) (PUFAs) (Table 2, column 4). The dicarboxylate and monohydroxy derivatives of some of these fatty acids were also increased (S1 Table, column Q rows 266–283 and rows 310–326), along with monoacylglycerols and many species of phosphatidylcholine (Table 2, column 4). These results are consistent with reports by others of differences in fatty acid metabolism in GF versus CONV mice [39–41]. Reductions in glucose with antibiotics are thought to result from increased glucose use by enterocytes which compensate for loss of SCFAs, their primary energy source, by increasing glycolysis [42]. Similarly, loss of energy derived from SCFAs in antibiotic-treated mice may lead to lipolysis and consequent increases in circulating lipids to provide fuel for energy production from fatty acid oxidation.

**Table 2:** Effect of ozone and antibiotics on serum lipids.

Lipid	Ozone/Air Water	Ozone/Air Antibiotics	Antibiotics/Water Air	Antibiotics/Water Ozone
**Medium chain fatty acids**				
caproate (6:0)	1.22*	1.11	1.36#	1.24#
heptanoate (7:0)	1.16*	0.75*	1.52#	0.98
caprylate (8:0)	1.24*	0.88	1.24#	0.88
pelargonate (9:0)	1.21	0.79	1.18	0.77
caprate (10:0)	1.24*	0.83	1.19	0.80#
undecanoate (11:0)	1.17	0.74*	1.46#	0.92
10-undecenoate (11:1n1)	1.53*	0.48*	4.10#	1.29
Laurate	1.32*	0.77*	1.43#	0.83
**Long chain fatty acids**				
myristate (14:0)	1.06	0.59*	1.30	0.73
myristoleate (14:1n5)	1.74*	0.64*	1.72#	0.63#
pentadecanoate (15:0)	0.92	0.63*	1.12	0.77#
palmitate (16:0)	0.87	0.61*	1.11	0.77
palmitoleate (16:1n7)	1.23	0.62	1.15	0.58#
margarate (17:0)	0.95	0.46*	1.67#	0.81
10-heptadecenoate (17:1n7)	1.03	0.54*	1.28	0.67#
stearate (18:0)	0.83	0.56*	1.35	0.91
oleate (18:1n9)	0.89	0.64*	1.18	0.85
cis-vaccenate (18:1n7)	0.84	0.74	1.10	0.97
nonadecanoate (19:0)	0.57*	0.46*	1.10	0.88
10-nonadecenoate (19:1n9)	1.07	0.49*	1.55	0.71
arachidate (20:0)	0.34*	0.36*	0.77	0.83
eicosenoate (20:1)	0.65	0.41*	1.05	0.67
erucate (22:1n9)	0.35*	0.31*	0.81	0.72
**PUFAs**				
stearidonate (18:4n3)	0.71	0.33*	1.49#	0.68#
eicosapentaenoate (EPA; 20:5n3)	1.27	0.64*	1.85#	0.93
docosapentaenoate (n3 DPA; 22:5n3)	1.07	0.50*	1.44	0.68#
docosahexaenoate (DHA; 22:6n3)	1.16	0.58*	1.67#	0.83
linoleate (18:2n6)	1.25	0.58*	1.46#	0.67#
linolenate [alpha or gamma; (18:3n3 or 6)]	1.07	0.41*	1.54#	0.59#
dihomo-linolenate (20:3n3 or n6)	0.82	0.63*	1.14	0.87
arachidonate (20:4n6)	1.27	1.11	1.53#	1.33#
adrenate (22:4n6)	0.81	0.49*	1.49	0.90
docosapentaenoate (n6 DPA; 22:5n6)	0.68*	0.47*	1.39#	0.96
docosadienoate (22:2n6)	0.40*	0.39*	0.71	0.69
dihomo-linoleate (20:2n6)	0.87	0.42*	1.42	0.68
mead acid (20:3n9)	0.78	0.71	1.16	1.06
**Monoacylglycerides**				
1-pentadecanoylglycerol (15:0)	0.55*	0.42*	1.25	0.97
1-palmitoylglycerol (16:0)	0.59*	0.31*	2.02#	1.06
2-palmitoylglycerol (16:0)	0.79	0.30*	2.49#	0.96
1-stearoylglycerol (18:0)	0.83	0.79	1.17	1.12
1-oleoylglycerol (18:1)	0.46*	0.42*	1.13	1.02
2-oleoylglycerol (18:1)	0.56*	0.36*	1.57#	0.99
1-linoleoylglycerol (18:2)	0.81	0.37*	1.85#	0.85
2-linoleoylglycerol (18:2)	0.65*	0.37*	1.48#	0.83
1-linolenoylglycerol (18:3)	0.43*	0.31*	1.09	0.79
1-arachidonylglycerol (20:4)	1.42*	0.49*	3.05#	1.05
2-arachidonoylglycerol (20:4)	0.98	0.65*	1.98#	1.31
1-docosahexaenoylglycerol (22:6)	0.77	0.48*	1.56#	0.97
1-dihomo-linolenylglycerol (alpha, gamma)	0.81	0.39*	1.93#	0.92
2-docosahexaenoylglcyerol	1.07	0.50*	2.07#	0.97
**Phosphatidylcholine**				
palmitoyl-arachidonoyl-glycerophosphocholine (1)	1.17*	1.05	1.12	1.01
palmitoyl-arachidonoyl-glycerophosphocholine (2)	1.19*	1.17*	1.03	1.01
palmitoyl-linoleoyl-glycerophosphocholine (1)	0.85*	0.84*	1.03	1.01
palmitoyl-linoleoyl-glycerophosphocholine (2)	0.85*	0.88*	0.98	1.01
palmitoyl-oleoyl-glycerophosphocholine (1)	0.74*	0.89	0.81#	0.97
stearoyl-arachidonoyl-glycerophosphocholine (1)	1.60*	1.03	1.66#	1.08
stearoyl-arachidonoyl-glycerophosphocholine (2)	1.57*	1.21*	1.38#	1.06
oleoyl-linoleoyl-glycerophosphocholine (1)	0.89*	0.93	1.12#	1.16#
oleoyl-linoleoyl-glycerophosphocholine (2)	0.89	0.83*	1.21#	1.13
palmitoyl-palmitoyl-glycerophosphocholine (1)	1.01	0.93	1.08	1.00
palmitoyl-palmitoyl-glycerophosphocholine (2)	1.14*	1.02	1.15#	1.03
stearoyl-linoleoyl-glycerophosphocholine (1)	0.78*	0.73*	1.26#	1.19#
stearoyl-linoleoyl-glycerophosphocholine (2)	0.76*	0.66*	1.37#	1.19#

For each metabolite, peaks were quantified using area-under-the-curve. Then, the median area-under-the-curve for that metabolite across all mice in all groups was calculated. Then, a scaled intensity for each metabolite was calculated by dividing the raw area counts for each mouse by this median so that the median across all mice in all groups was now equal to 1. A two way ANOVA using the factors, treatment and exposure, was then performed on log transformed data. Follow-up pairwise contrasts were performed using F-tests. Storey’s q-values were calculated to estimate the proportion of false positives. Results here are the ratio of mean lipid metabolite scaled intensity in O_3_ versus air exposed mice treated with regular drinking water (column 2) or in O_3_ versus air exposed mice treated with antibiotic-supplemented drinking water (column 3). Also shown are the mean lipid metabolite scaled intensity in antibiotic versus water treated mice exposed to air (column 4) or O_3_ (column 5). For example, a ratio of 1.5 for a metabolite indicates a 50% increase in that metabolite. Green and red highlighted areas indicate a significant (p<0.05 and q<0.10) decrease or increase in that metabolite.

*p<0.05 and q<0.10 versus air exposed mice with the same treatment;

^#^ p<0.05 and q<0.10 versus water-treated mice with the same exposure.

S1 Table contains these p and q values for each metabolite. n = 8/group.

**Fig 3:**
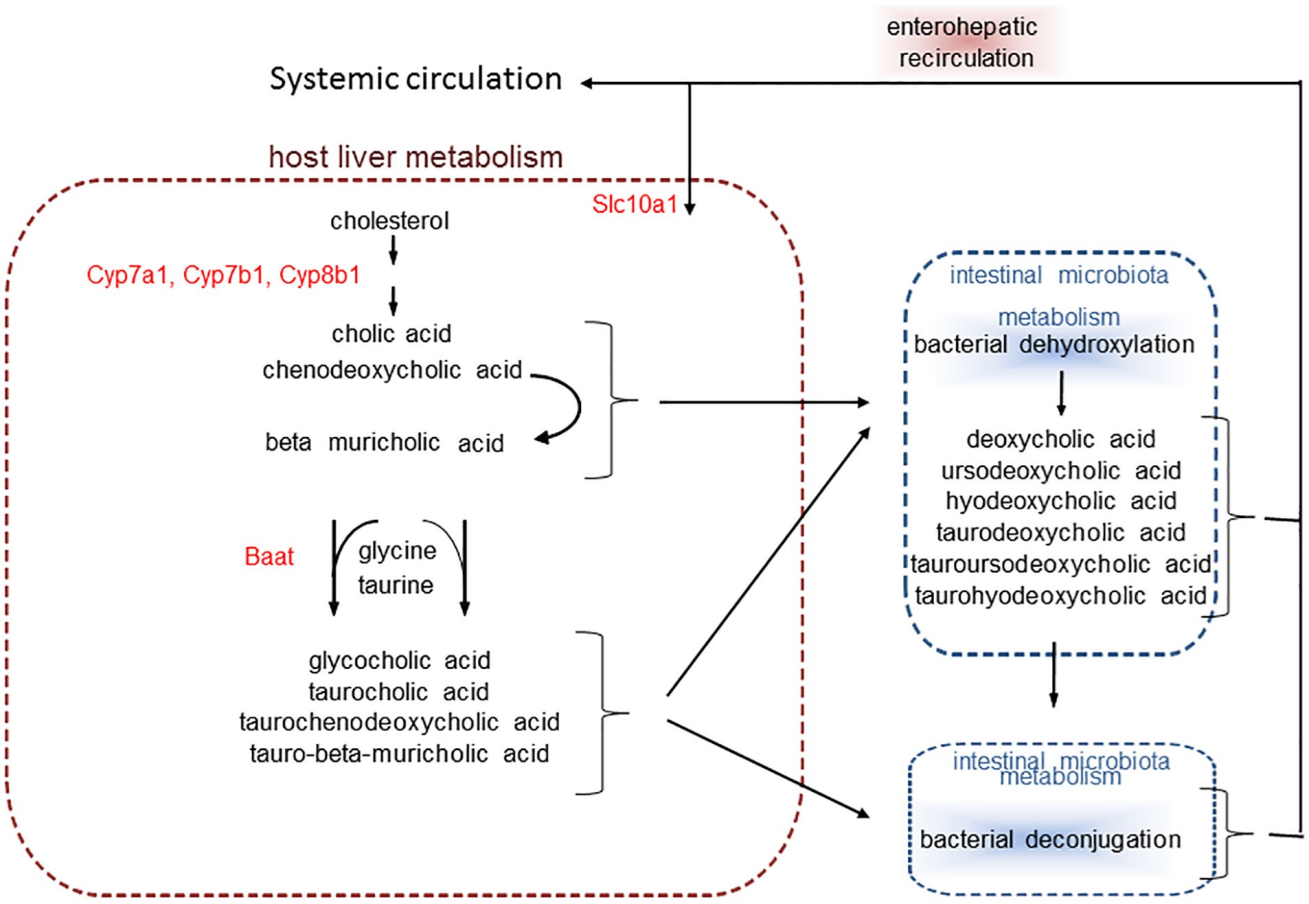
Schematic representation of bile acid synthesis and bacterial metabolism. Bacterial processes that modify bile acids are indicated by the dashed blue boxes. The primary bile acids cholic acid, chenodeoxyholic acid, and β muricholic acid are produced from cholesterol, conjugated with taurine or glycine in the host liver, and secreted into the bile. In the intestinal lumen, bacteria convert primary bile acids to secondary bile acids by dehydroxylation and also deconjugate bile acids. Bile acids are absorbed across enterocytes into the enterohepatic circulation and returned to the liver. A portion (about 5%) remain in the blood and are delivered to the systemic circulation. Liver expression of the enzymes indicated in red text were assayed.

In mice exposed to air, there were also effects of antibiotics on serum bile acids (Table 3, column 4). In mice, the primary bile acids cholate, chenodeoxycholate, and β-muricholate, are synthesized from cholesterol, conjugated with taurine and to a lesser extent, glycine, in the liver, and secreted into the duodenum where they participate in the emulsification of dietary fat [43, 44] (Fig 3). Most bile acids are absorbed back into the circulation in the lower gastrointestinal (GI) tract, and returned to the liver (enterohepatic recirculation) (Fig 3). However, a portion of bile acids escape re-uptake and circulate in the systemic blood [43, 45] as observed (Table 3). Gut bacteria deconjugate bile acids. Gut bacteria also dehydroxylate bile acids resulting in the formation of secondary bile acids [44] (Fig 3). Consequently, antibiotic treatment should increase most conjugated bile acids, and decrease most secondary bile acids as observed (Table 3, column 4), though cholesterol itself was not affected by antibiotic treatment (Table 3). These results are consistent with reports by others in both antibiotic-treated and GF mice [46, 47].

**Table 3:** Effect of ozone and antibiotics on serum bile acids.

Bile acid	Ozone/Air Water	Ozone/Air Antibiotics	Antibiotics/Water Air	Antibiotics/Water Ozone
**Primary bile acids**				
cholate	0.10*	5.29*	0.03#	1.85
glycocholate	0.79	5.70*	0.68	4.92#
taurocholate	0.77	11.43*	2.31	34.14#
chenodeoxycholate	0.73	1.66	0.99	2.27#
taurochenodeoxycholate	1.24	10.99	5.18#	45.98#
beta muricholate	0.26	19.05*	0.08#	5.97#
tauro-beta-muricholate	0.54	22.72*	3.58#	150.95#
**Secondary bile acids**				
deoxycholate	1.12	10.91*	0.07#	0.66#
taurodeoxycholate	2.44	17.12*	1.06	7.42
ursodeoxycholate	0.09*	4.53*	0.05#	2.45
tauroursodeoxycholate	0.62	36.50*	2.37	138.75#
hyodeoxycholate	0.63	1.62	0.18#	0.46
taurohyodeoxycholate	1.57	22.74*	0.64	9.25#
cholesterol	0.82*	0.94	1.00	1.15

Results are the ratio of mean bile acid scaled peak area in mice treated with regular drinking water or antibiotic-supplemented drinking water and exposed to air or ozone. See figure legend for Table 2 for details.

*p<0.05 and q<0.10 versus air exposed mice with the same treatment;

^#^ p<0.05 and q<0.10 versus water treated mice with the same exposure.

Green indicates a significant decrease and red indicates a significant increase. n = 8/group

**Fig 4:**
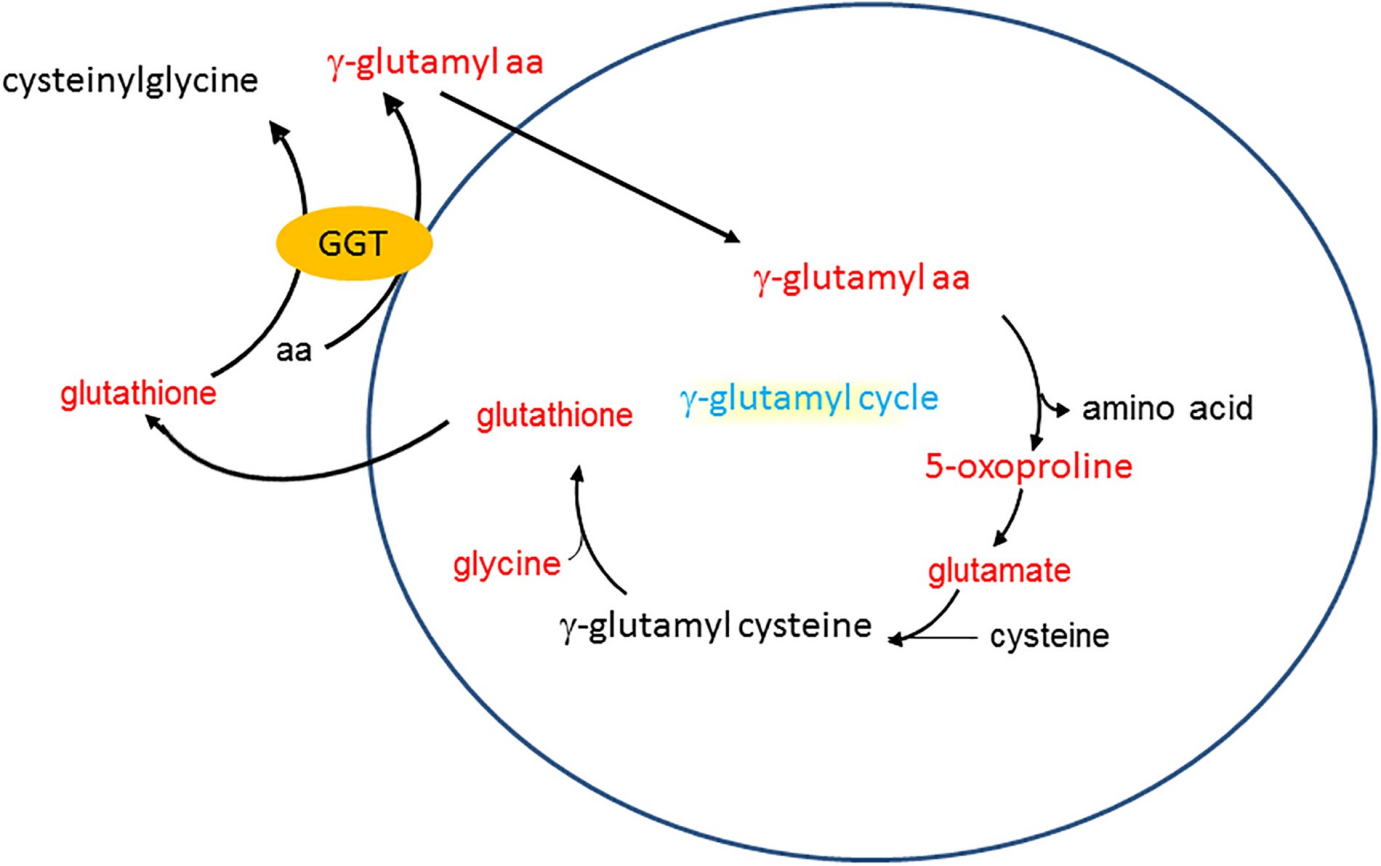
Schematic representation of the γ-glutamyl cycle. Gamma glutamyl transferase (GGT) metabolizes glutathione, in the presence of an amino acid, to form a γ-glutamyl amino acid and cysteinylglycine. γ-glutamyl amino acids are then metabolized to oxoproline and an amino acid and oxoproline is converted back to glutathione. Metabolites in red were significantly affected either by O_3_ or antibiotics or both.

There was a marked effect of antibiotic treatment on serum γ-glutamyl amino acids: all 12 of the12 γ-glutamyl amino acids identified in serum were decreased in antibiotic-treated versus control mice exposed to air (Table 4, column 4). Others have reported *increases* in *fecal* γ-glutamyl amino after administration of the antibiotic, clindamycin, in mice [48], likely because intestinal bacteria no longer metabolize and degrade these metabolites. Thus, the *decreases* in *serum* γ-glutamyl amino acids that we observed in antibiotic-treated mice are unlikely to be the result of reduced delivery of these metabolites to the serum from the intestines. γ-glutamyl amino acids are produced by the enzyme gamma-glutamyl transferase (GGT) which transfers the gamma-glutamyl moiety of glutathione to amino acids (Fig 4). GGT activity is a marker of oxidative stress [49]. Consequently, the data suggest reduced oxidative stress in the antibiotic- versus water-treated mice. To our knowledge, this is the first report of alterations in serum γ-glutamyl amino acids in either antibiotic-treated or GF mice. However, Mardinoglu et al [50] also noted changes in glutathione metabolism in liver and intestinal tissues of GF versus CONV mice, consistent with a role for the microbiome in the regulation of this metabolic pathway.

**Table 4:** Effect of ozone and antibiotics on serum γ-glutamyl amino acids.

γ-glutamyl amino acids	Ozone/Air Water	Ozone/Air Antibiotics	Antibiotics/Water Air	Antibiotics/Water Ozone
γ -glutamylalanine	0.60*	0.84	0.65#	0.91
γ -glutamylglutamate	0.26*	0.40*	0.48#	0.73
γ -glutamylglutamine	0.62*	1.16	0.40#	0.76#
γ -glutamylisoleucine	0.52*	0.57*	0.70#	0.76#
γ -glutamylleucine	0.50*	0.63*	0.61#	0.77#
γ -glutamyl-epsilon-lysine	0.51*	0.58*	0.70#	0.80
γ -glutamylmethionine	0.46*	0.93	0.54#	1.09
γ -glutamylphenylalanine	0.70*	0.73*	0.77#	0.80#
γ -glutamylthreonine	0.65*	0.82*	0.66#	0.84
γ -glutamyltryptophan	0.60*	0.65*	0.79#	0.85
γ -glutamyltyrosine	0.54*	0.63*	0.67#	0.78
γ -glutamylvaline	0.53*	0.59*	0.64#	0.72#

Results are the ratio of mean γ-glutamyl amino acid scaled peak area in mice treated with regular drinking water or antibiotic-supplemented drinking water and exposed to air or ozone. See figure legend for Table 2 for details.

*p<0.05 and q<0.10 versus air exposed mice with the same treatment;

^#^ p<0.05 and q<0.10 versus water treated mice with the same exposure.

Green indicates a significant decrease. n = 8/group

### Effect of O_3_ exposure on the serum metabolome of water-treated mice

**Fig 5:**
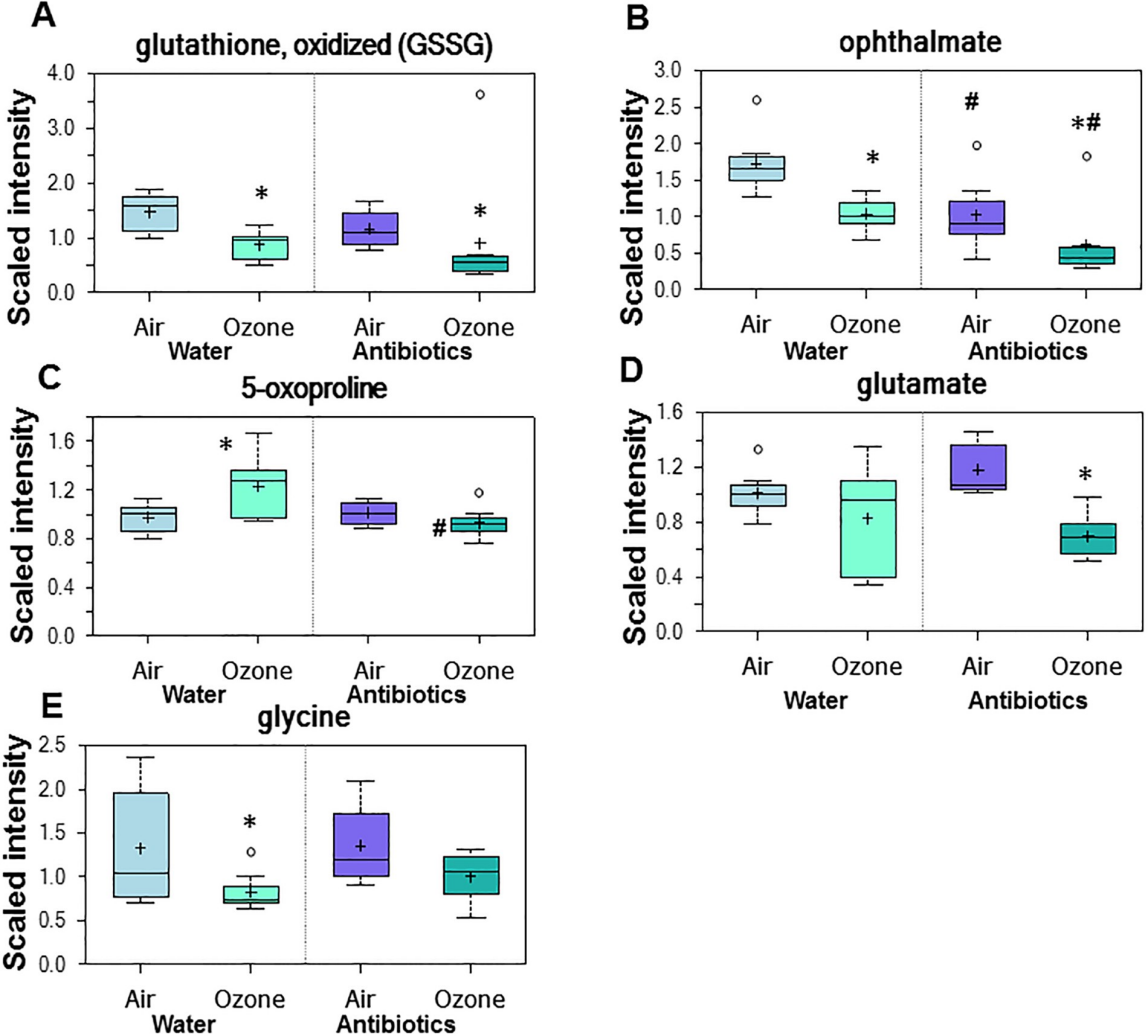
Serum oxidized glutathione (A) and ophthalmate (B) and other metabolites in the γ-glutamyl pathway (C-E) in mice treated with water or antibiotics prior to air or ozone. Results are expressed as described in Fig 2. n = 8/group * p<0.05 and q<0.10 versus air exposed mice with the same treatment. # p<0.05 and q<0.10 versus water-treated mice with the same exposure.

To determine whether there were metabolic pathways that were enriched among those metabolites significantly affected by O_3_ exposure, we calculated an enrichment factor (Table 5) for each superpathway and for pathways within each superpathway (see Methods). Metabolic pathway enrichment analysis indicated effects of O_3_ exposure on γ-glutamyl amino acids in serum from mice treated with regular drinking water (Table 5). All 12 of the 12 γ-glutamyl amino acids detected in serum were reduced by about half in mice exposed to O_3_ (Table 4, column 2). As discussed above, decreases in circulating γ-glutamyl amino acids may be the result of reduced expression of GGT reflecting reduced oxidative stress. Serum levels of oxidized glutathione were also reduced following O_3_ exposure in water-treated mice (Fig 5A) suggesting reduced oxidative stress. Reduced glutathione was not among the biochemicals identified on the metabolomics array, so we cannot determine whether the lower levels of oxidized glutathione were the result of lower total glutathione. However, the observation that ophthalmate, a marker of oxidative stress [51], was also reduced by O_3_ exposure (Fig 5B), is consistent with reduced oxidative stress. O_3_ exposure induces expression of a variety of antioxidant enzymes [25, 31], which may account for the apparent reduction in oxidative stress. O_3_-induced changes in other components of the γ-glutamyl pathway (Fig 4) that were identified in our array are shown in Fig 5C–5E.

**Table 5:** Metabolic pathways affected by ozone exposure in water- and antibiotic-treated mice.

Superpathway	Pathway	n	Water-treated		Antibiotic-treated	
			EF	p value	EF	p value
Amino acids		163	1.11	0.24	0.90	0.12
Peptides		20	**1.53**	0.033	1.16	0.454
	γ-glutamyl amino acids	12	**2.19**	0.0002	1.45	0.104
Carbohydrates		27	0.73	0.206	0.86	0.434
Energy		8	0.55	0.24	0.97	0.919
Lipids		217	0.97	0.708	**1.18**	0.0003
	Medium chain fatty acid	8	1.64	0.099	0.97	0.919
	Long chain fatty acid	15	0.58	0.133	**1.67**	0.0061
	Polyunsaturated fatty acid	13	**0.34**	0.030	**1.63**	0.0165
	Fatty acid, dicarboxylate	16	**0.41**	0.032	1.45	0.0593
	Monohydroxy fatty acid	17	**0.39**	0.022	1.25	0.279
	Phospholipid metabolism	6	0.36	0.155	0.97	0.930
	Lysolipid	30	1.38	0.060	1.03	0.861
	Phosphatidylcholine	13	**1.85**	0.0055	1.04	0.880
	Other phospholipids (PE, PI, and glycerolipid metabolism)	9	1.70	0.056	1.07	0.819
	Monoacylglyceride	14	1.09	0.75	**1.79**	0.0018
	Sphingolipid metabolism	9	1.46	0.21	1.29	0.368
	Bile acid metabolism	13	**0.34**	0.030	1.49	0.0664
Nucleotides		41	**0.64**	0.040	0.85	0.295
Cofactors and vitamins		23	1.14	0.54	0.76	0.215
Xenobiotics		63	1.07	0.60	0.80	0.077

n: number of metabolites in pathway; Enrichment factor (EF) was computed as follows: (# of metabolites in pathway significantly affected by ozone exposure/ total # of detected metabolites in pathway)/ (total # of metabolites affected by ozone/total # of detected metabolites). Individual metabolites were considered to have been significantly affected if p<0.05 and q<0.10. p values in the table indicate the significance of enrichment of the metabolite group compared to the total number of significantly affected metabolites and were computed by Chi square test. Pathways with significant enrichment of significantly altered metabolites are indicated in bold text.

As indicated in Table 5, phosphatidylcholine metabolites were also significantly enriched among the metabolites affected by O_3_ (see also Table 2, column 2). Reductions in lung phosphatidylcholine are also observed following O_3_ exposure, likely because of ozonization of these metabolites [52]. O_3_ also caused significant changes in metabolites within other lipid pathways. For example, 6 of 8 medium chain fatty acids increased in the serum after O_3_ exposure (see also Table 2, column 2). O_3_ exposure also caused reductions in many serum lysolipids (S1 Table, column O, rows 346–375) and monoacylglycerides (Table 2, column 2). The reductions in lysolipids are consistent with the changes observed in the lung after O_3_ exposure [25].

**Fig 6:**
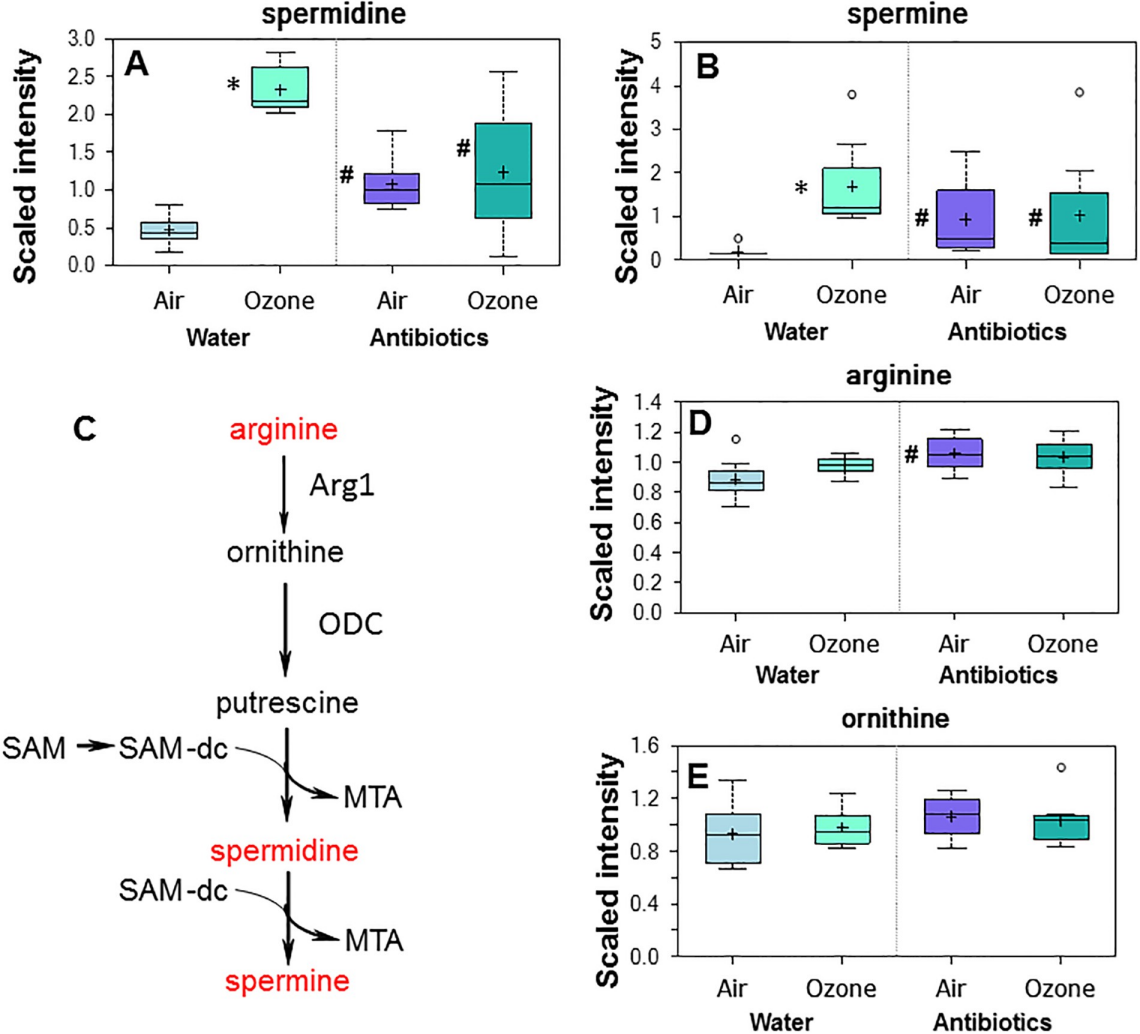
Effect of ozone exposure and antibiotics on serum polyamines. Serum (A) spermidine and (B) spermine in mice treated with water or antibiotics prior to air or ozone. (C) Schematic representation of the metabolism of polyamines. Arg1: arginase 1; ODC: ornithine decarboxylase; SAM: S-Adenosinemethionine; SAM-dc: Decarboxy-S-adenosinemethionine; MTA: 5-methylthioadenosine (MTA). In C, metabolites that were affected by antibiotics, ozone, or the combination of antibiotics and ozone are shown in red. Results are expressed as described in Fig 2. n = 8/group * p<0.05 and q<0.10 versus air exposed mice with the same treatment. # p<0.05 and q<0.10 versus water-treated mice with the same exposure.

There were also changes in other metabolites following O_3_ exposure. O_3_ changed many serum amino acids and their metabolites, consistent with data reported in rats [23]. Among these amino acid metabolites, we noted substantial (8.6- and 10.6-fold, respectively) and significant increases in p-cresol sulfate and p-cresol glucuronide (S1 Table, column O, rows 64 and 79). These biochemicals are generated by dietary tyrosine transformation by gut bacteria followed by sulfation and glucuronidation in the liver. Thus, increases in p-cresol sulfate and p-cresol glucuronide following O_3_ exposure may reflect either alterations in the gut microbiome following O_3_ exposure, or altered processing of these biochemicals as a result of O_3_-induced changes in liver enzymes. O_3_-induced changes in gene expression within the liver have been reported by others [23, 53]. We also observed significant and substantial (9-fold and 5-fold) increases in the polyamines, spermine and spermidine, after O_3_ exposure (Fig 6A and 6B). Spermidine and spermine are synthesized from ornithine, an amino acid produced from arginine as part of the urea cycle [54](see Fig 6C). However, neither ornithine nor arginine was impacted by O_3_ (Fig 6D and 6E). Increases in polyamines are also observed in the lungs of rats after exposure to O_3_ (0.5 ppm continuously for 5 days) [55]. Whether the changes we observed in the serum following O_3_ (Fig6A and 6B) are the result of uptake from the lung remains to be established. Decreases in several plant-derived food components were also observed in serum after O_3_ exposure (S1 Table, column O, lines 521–551). In rodents, O_3_ exposure causes a decline in metabolic rate and a corresponding reduction in food intake [56] followed by recovery. Hence, it is possible that reductions in these plant-derived food components reflect altered food consumption after O_3_.

### Impact of antibiotics on O_3_-induced changes in the serum metabolome

As discussed above, γ-glutamyl amino acids and phosphatidylcholine species were significantly enriched among the metabolites affected by O_3_ in water-treated mice (Table 5). O_3_-induced changes in γ-glutamyl amino acids and phosphatidylcholine metabolites were qualitatively similar in water-treated and antibiotic treated mice, although the changes reached statistical significance for fewer of these metabolites in antibiotic-treated than water-treated mice (Tables 2 and 4). In contrast, the impact of O_3_ on other lipids was markedly different in control and antibiotic-treated mice (Table 2). Whereas O_3_ increased most medium chain fatty acids in control mice (Table 2, column 2), O_3_ caused significant reductions in 4 of 8 medium chain fatty acids in antibiotic treated mice (Table 2, column 3). In antibiotic-treated mice, 13 of 15 long chain fatty acids, and 11 of 13 polyunsaturated fatty acids (PUFAs) were also reduced by O_3_ exposure (Table 2, column 3), whereas only 4 of 15 long chain fatty acids and 2 of 13 PUFAs were affected by O_3_ exposure in water-treated mice (Table 2, column 2). Among the metabolites affected by O_3_ exposure, enrichment factor analysis indicated a significant enrichment in these metabolite groups along with monoacylglycerides in the antibiotic-treated but not water-treated mice (Table 5).

Many tyrosine and tryptophan metabolites were also differentially affected by O_3_ exposure in control and antibiotic treated mice, likely because many of these metabolites are derivatives of bacterial metabolism of these amino acids (S1 Table, compare columns O and P, rows 52–98). The marked increases in the polyamines, spermine and spermidine, observed after O_3_ exposure in control mice were absent in antibiotic treated mice (Fig 6A and 6B).

**Fig 7:**
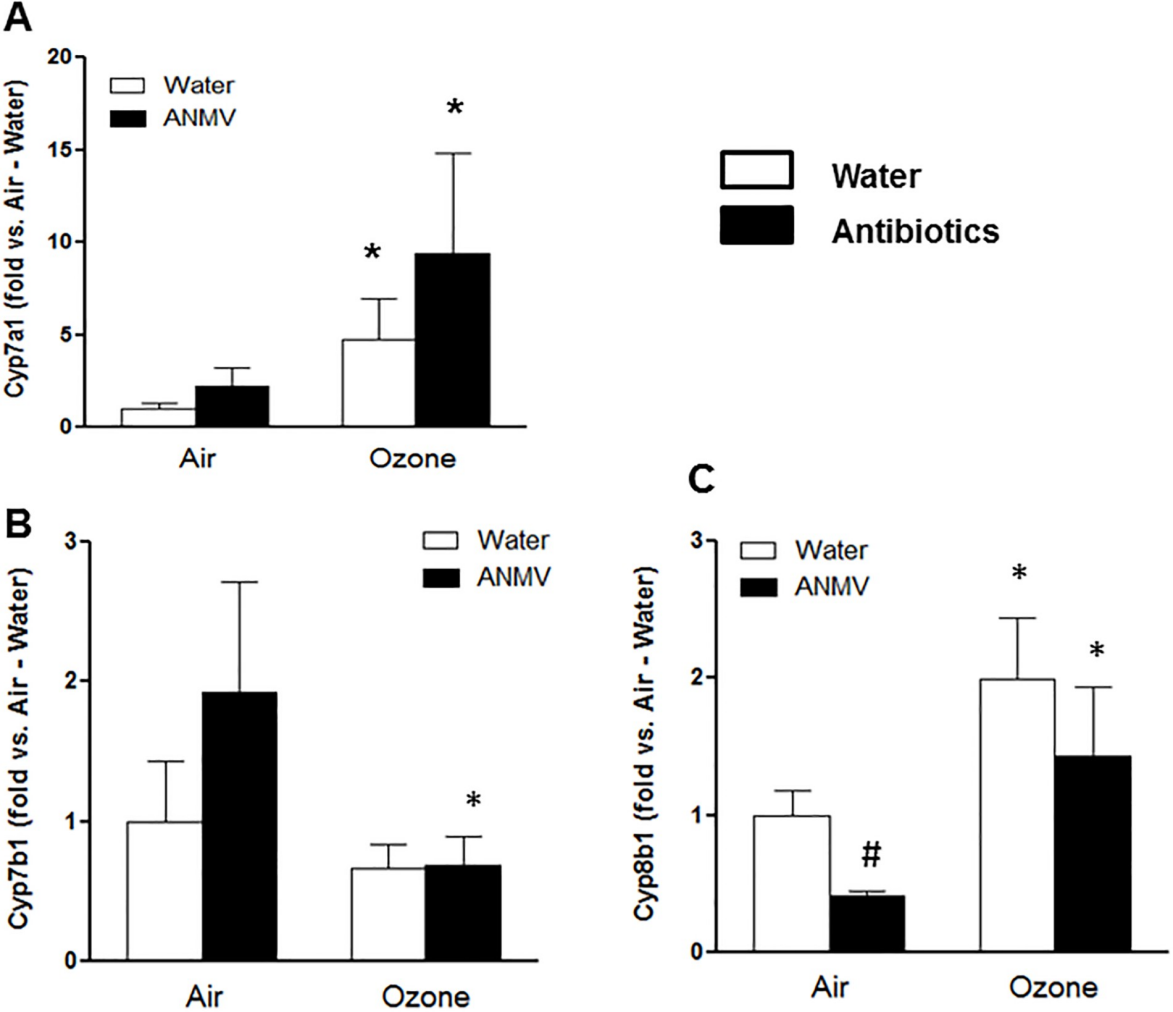
Liver mRNA abundances of genes involved in bile acid synthesis in mice treated with water or antibiotics prior to air or ozone. Results are expressed relative to expression in air-exposed water-treated mice and are shown as mean +/- SE of data from n = 6-7/mice per group * p<0.05 versus air exposed mice with the same treatment. # p<0.05 versus water-treated mice with the same exposure.

O_3_-induced changes in serum bile acid metabolites also differed substantially in water- versus antibiotic-treated mice (Table 3, columns 2 and 3). Of the 13 detected bile acids, only 2 (cholate and ursodeoxycholate) were significantly decreased in serum of O_3_- versus air-exposed mice that had been treated with water (Table 3, column 2), whereas 10 of the 13 bile acids were significantly increased O_3_- versus air-exposed mice treated with antibiotics (Table 3, column 3). These changes were not only significant, but also substantial in magnitude. Others have reported changes in expression of some liver enzymes involved in bile acid metabolism following O_3_ exposure in rats [23, 53]. To determine whether the marked effects of antibiotic treatment on serum bile acids observed in O_3_ exposed mice might be the result of effects of O_3_ and/or antibiotic treatment on the liver’s ability to synthesize, conjugate, excrete, or take up recirculating bile acids, we measured liver mRNA abundances of *Cyp7a1*, *Cyp7b1*, *Cyp8b1*, *Baat*, and *Slc10a1*. Conversion of cholesterol to 7-α-hydroxycholesterol by *Cyp7a1* is both the first and the rate limiting step in the generation of bile acids [57]. Compared to air, exposure to O_3_ caused a significant (p<0.002) increase in the mRNA abundance of *Cyp7a1* that was observed in both antibiotic- and water-treated mice, but there was no significant effect of antibiotic treatment (Fig 7A). *Cyp7b1* is part of an alternative pathway for conversion of cholesterol to bile acids. In mice, this pathway contributes to about 25% of bile formation. Factorial ANOVA also indicated a significant (p<0.05) effect of O_3_ exposure on liver *Cyp7b1* expression. Follow up analysis indicated that the effect of O_3_ on *Cyp7b1* expression lay in the antibiotic-treated mice, in which O_3_ decreased *Cyp7b1* expression, whereas there was no significant effect of O_3_ was observed in the water treated mice (Fig 7B). *Cyp8b1* catalyzes the conversion of 7 alpha-hydroxy-4-cholesten-3-one into 7-alpha,12-alpha-dihydroxy-4-cholesten-3-one, an event that produces cholate instead of chenodeoxycholate. There was a significant effect of both O_3_ exposure (p<0.001) and antibiotic treatment (p<0.02) on liver *Cyp8b1* mRNA abundance. O_3_ exposure significantly increased liver *Cyp8b1* expression in both water and antibiotic treated mice (Fig 7C). In addition, antibiotic treatment significantly decreased *Cyp8b1* in air- but not O_3_-exposed mice (Fig 7C). Other liver enzymes involved in bile acid conjugation and reuptake in the liver, including *Baat*, and *Slc10a1*, were not affected by either O_3_ exposure or antibiotic treatment. The function of these enzymes is presented schematically in Fig 3.

### Effect of O_3_ exposure and antibiotic treatment on hormones affecting metabolism

**Fig 8:**
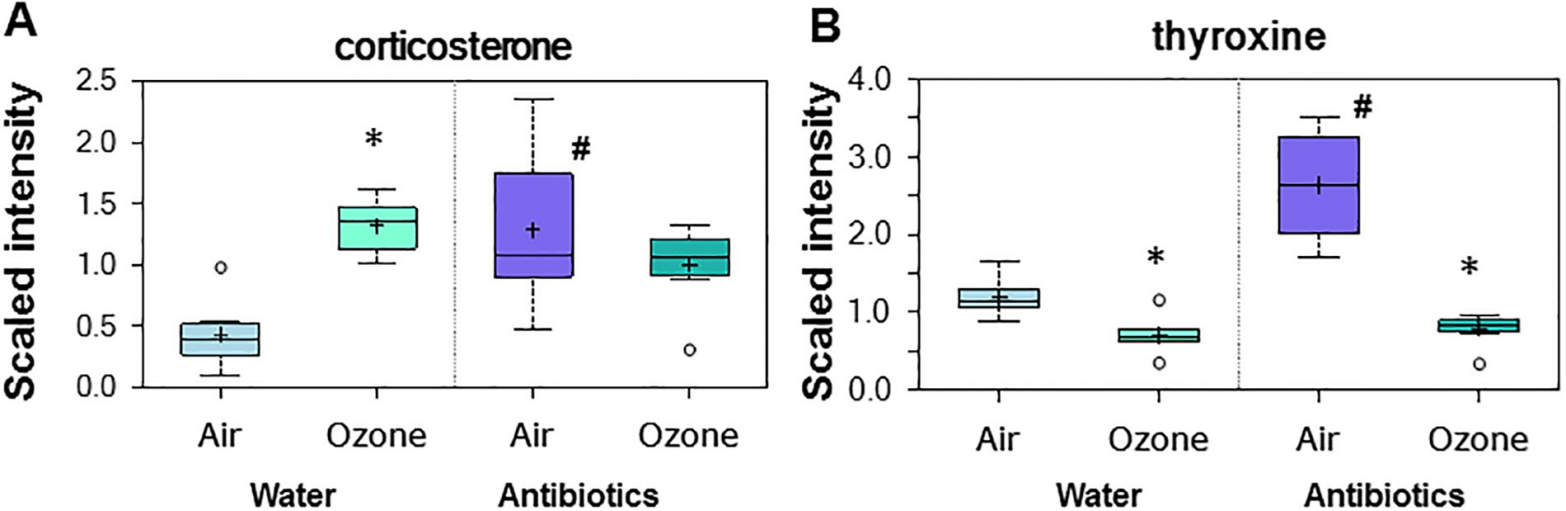
Serum corticosterone (A) and thyroxine (B) in mice treated with water or antibiotics prior to air or ozone. Results are expressed as described in Fig 2. n = 8/group * p<0.05 and q<0.10 versus air exposed mice with the same treatment. # p<0.05 and q<0.10 ersus water-treated mice with the same exposure.

In rodents, acute exposure to O_3_ reduces thyroxine and increases corticosterone [25, 58, 59] consistent with the overall reduction in metabolic rate and the increased lipolysis typically observed in these animals immediately after O_3_ exposure [23, 60]. Because of the marked differences in the effect of O_3_ on lipid metabolites in control versus antibiotic-treated mice (Tables 2 and 5), we examined serum concentrations of thyroxine and corticosterone (Fig 8). In air-exposed mice, antibiotic treatment caused a significant increase in both thyroxine and corticosterone (Fig 8). In control (water-treated) mice, exposure to O_3_ caused a decrease in thyroxine and an increase in corticosterone, consistent with previous reports [25, 58, 59] in rodents. In antibiotic-treated mice, O_3_ still caused a marked reduction in serum thyroxine (Fig 8B). However, in contrast to the effects of O_3_ on corticosterone in control mice, O_3_ had no effect on serum corticosterone in antibiotic-treated mice (Fig 8A). The increases in serum corticosterone in air-exposed mice treated with antibiotics (Fig 8A) are consistent with studies demonstrating effects of the gut microbiome on hypothalamic-anterior pituitary (HPA) activity. For example, compared to conventionally raised rodents, rodents raised under germ-free (GF) conditions have increased activity of the HPA axis in response to acute stressors like restraint [61, 62]. Bacteria have been shown to bind to and degrade thyroxine [63] which may explain the observed increases in serum thyroxine (Fig 8B) that occurred in air-exposed mice after gut bacteria were depleted by antibiotic treatment. However, it is also possible that antibiotic-related increases in serum thyroxine are the result of alterations in the activation of the HPA axis leading to greater synthesis of thyroid stimulating hormone.

## Discussion

The purpose of this study was to identify metabolites affected by the gut microbiome that may mediate the role of the microbiome in pulmonary responses to O_3_ [1]. Exposure to O_3_ changed many circulating metabolites in both water-treated and antibiotic-treated mice (Table 1 and S1 Table). For many of these metabolites, the effect of O_3_ was similar in control and antibiotic-treated mice. However, exposure to O_3_ had substantially different effects on many serum lipids in water- and antibiotic-treated mice. In particular, O_3_ decreased serum concentrations of many long chain fatty acids and PUFAs and increased serum concentrations of many bile acid species in antibiotic- but not water-treated mice. O_3_ also caused substantial increases in serum concentrations of the polyamines, spermine and spermidine, in water- but not antibiotic-treated mice. As discussed below, changes in these metabolites may explain the observed protective effect of germ-free conditions or antibiotic treatment on O_3_-induced AHR and neutrophilic inflammation [1].

Even in air-exposed mice, antibiotic treatment had a substantial effect on the serum metabolome. Nearly 45% of the metabolites identified were significantly different in control versus antibiotic-treated mice exposed to air (Table 1). The metabolites that were altered by antibiotics are similar to those reported by others to be affected by the gut microbiome [17, 18]. The data thus provide a good positive control indicating the efficacy of the antibiotic treatment. Many of the metabolites that decreased with antibiotic treatment were metabolites of known bacterial origin (e.g. propionylcarnitine, butyrlcarnitine, equol, indole propionate, 3-indoxyl sulfate, and 3-(4-hydroxyphenyl)propionate (Fig 2)) that are produced from dietary substrates [18, 36–38]. Reductions in these metabolites likely reflect depletion of gut microbiota, reductions in the metabolites they produce, and consequent reductions in the transit of these metabolites across enterocytes to the blood. Other metabolites that decreased with antibiotic treatment in air-exposed mice are not produced by bacteria but are host-derived biochemicals that are enzymatically modified by gut bacteria (e.g. secondary bile acids (Table 2)). Depletion of bacteria by antibiotic treatment prevents generation of these metabolites. Other metabolites that decreased with antibiotic treatment are generated in the liver from bacterial-derived precursors, as described by others [18]. We also observed increases in some serum metabolites with antibiotic treatment. Many of these metabolites are diet-derived biochemicals normally metabolized by gut bacteria. For example, preventing bacterial generation of equol (Fig 2) from dietary daidzein by antibiotic treatment resulted in increased daidzein (S1 Table, column Q, row 529). Preventing bacterial generation of indoles (Fig 2) from dietary tryptophan with antibiotic treatment resulted in increased tryptophan (S1 Table, column Q, row 83). Increases in other serum metabolites with antibiotic treatment likely occurred as a result of changes in substrates used for host metabolism, as described in Results.

In antibiotic-treated mice, O_3_ caused significant decreases in most long chain fatty acids (Table 2). In contrast, in water-treated mice, O_3_ caused only minimal changes in these metabolites. Airway smooth muscle expresses the G protein coupled receptor, FFAR1, which binds long chain fatty acids [64]. Moreover, long chain fatty acids increase intracellular calcium in cultured airway smooth muscle and augment acetylcholine-induced contraction of guinea pig airways [64]. Thus, reductions in long chain fatty acids in antibiotic-treated mice after O_3_ exposure would be expected to reduce airway responsiveness, as we have observed [1]. Neutrophils also express FFAR1, and undergo activation in response to long chain fatty acids [65]. Thus, it is conceivable that reductions in long chain fatty acids in antibiotic- versus water-treated mice exposed to O_3_ (Table 2) contribute to reductions in BAL neutrophils observed in these mice [1].

The reductions in serum long chain fatty acids, PUFAs, and monoglycerides observed after O_3_ exposure in antibiotic-treated mice (Tables 2 and 5) are consistent with reduced lipolysis in these mice. In contrast, in microbially-intact rodents, O_3_ exposure induces lipolysis with effects peaking immediately after O_3_ exposure and subsiding over the next 18–24 hours [23, 25]. Indeed, in water-treated mice, medium chain fatty acids and some long chain fatty acids increased after O_3_ exposure (Table 2), suggesting lipolysis. Antibiotic-dependent differences in the effects of O_3_ on serum corticosterone (Fig 8), which promotes lipolysis in adipose tissue, may explain these differences in O_3_-dependent lipolysis. In control mice, O_3_ caused an increase in serum corticosterone (Fig 8A), consistent with previous reports from ourselves and others [25, 58]. In contrast, O_3_ did not alter corticosterone in antibiotic-treated mice (Fig 8A). We also observed increases in serum corticosterone in air-exposed mice after antibiotic treatment (Fig 8A), an effect that should promote lipolysis, consistent with the elevated serum lipids observed in air-exposed antibiotic-treated mice (Table 2). The increases in serum corticosterone in air-exposed mice treated with antibiotics are consistent with studies demonstrating effects of the gut microbiome on hypothalamic-anterior pituitary (HPA) activity. For example, compared to conventionally raised rodents, rodents raised under germ-free conditions have increased activity of the HPA axis in response to acute stressors like restraint [61, 62].

O_3_ increased serum concentrations of many bile acids, especially taurine-conjugated bile acids, in antibiotic-treated but not water-treated mice (Table 3). In addition to their emulsifying actions, bile acids have a signaling role via their capacity to bind two receptors: farnesoid X receptor (FXR), a nuclear receptor, and TGR5, a G-protein coupled receptor. It is possible that elevations in bile acids after O_3_ exposure in antibiotic- treated mice (Table 3) contribute to reductions in O_3_-induced AHR and neutrophil recruitment to the lungs observed in antibiotic-treated mice [1]. For example, bile acids, promote relaxation of gastric smooth muscle via TGR5 activation of Gαs and consequent increases in cAMP [66]. Airway smooth muscle also relaxes in response to increases in cAMP, Bile acids also have TGR5-mediated anti-inflammatory effects [67–69]. Thus elevations in bile acids in antibiotic-treated mice exposed to O_3_ may also explain the reductions in neutrophil recruitment to the lungs observed in these mice [1].

We do not know the mechanistic basis for the increases in bile acids observed in antibiotic-treated mice exposed to O_3_ (Table 3). Analysis of liver gene expression indicated increases in *C7p7a1* and *Cyp8b1* in O_3_- versus air-exposed mice treated mice (Fig 7). Increases in the expression of these enzymes would be expected to promote bile acid synthesis, as observed (Table 3), but the effects on *C7p7a1* and *Cyp8b1* gene expression were also observed in water-treated mice (Fig 7), whereas no changes in serum bile acids were observed with O_3_ exposure in those mice (Table 3).

We observed marked increases in the polyamines, spermine and spermidine, in serum of water- but not antibiotic-treated mice after O_3_ exposure (Fig 6A and 6B). These polyamines are produced in most mammalian cells, but are also generated by gut bacteria from dietary arginine [16]. Thus, it is conceivable that reductions in spermine and spermidine in O_3_-exposed mice treated with antibiotics versus water (Fig 6A and 6B) reflect reductions in bacterial rather than host synthesis of these metabolites. Spermine increases cholinergic contraction of murine airways in vitro [70] and intratracheal administration of spermine causes bronchoconstriction [71]. Moreover, in mice, inhibition of polyamine synthesis attenuates allergen-induced AHR [71]. Thus, lower levels of spermine and spermidine in antibiotic- versus water-treated mice exposed to O_3_ (Fig 6A and 6B) would be expected to result in lower O_3_-induced AHR in the antibiotic-treated mice, as we have observed [1]. It is also conceivable that polyamines contribute to differences in O_3_-induced neutrophil recruitment to the lungs observed in water- versus antibiotic-treated mice [1]. Spermine levels are higher in sputum of patients with cystic fibrosis than in healthy controls [70], and there is a correlation between neutrophil recruitment to the lung and BAL polyamine levels in patients with cystic fibrosis [72]. It is interesting to note that high levels of polyamines are also present in serum of asthmatics during exacerbations [73]. Moreover, in patients with cystic fibrosis, elevations in sputum polyamines are attenuated after antibiotic treatment, similar to lower serum polyamines we observed in antibiotic-treated versus control mice exposed to O_3_ (Fig 6A and 6B).

There are several limitations to this study. First, we studied only male mice. There are sex differences in the effects of O_3_ on AHR [2, 74], and in the effects of the microbiome on pulmonary responses to O_3_ [74]. Hence, it is possible that there are also sex differences in the metabolomic response to O_3_ and its modulation by antibiotics. Second, we used a cocktail of antibiotics to deplete the microbiome. We and others have previously reported marked effects of this cocktail on the microbiome as assessed by 16S rRNA sequencing of fecal DNA [1] and our data indicate appropriate reductions in known microbial-derived metabolites after antibiotics (Fig 2). It is conceivable that there were off-target effects of the antibiotic cocktail that contributed to some of the observed antibiotic-dependent changes in the metabolomic response to O_3_. However, we have reported that in lean male mice, effects of antibiotics on O_3_-induced AHR are not observed in germ-free mice [1], indicating that the effects of antibiotics observed in conventional mice require an intact microbiome and hence are related to microbial depletion. In our studies of antibiotic- and ozone-induced changes in expression of genes related to bile acid metabolism (Fig 7), it is conceivable that antibiotics and/or O_3_ caused changes in protein expression independent of changes in gene transcription. Since we analyzed the liver and not the colon, it is also possible that there were unexamined changes in genes related to bile uptake across enterocytes. Finally, we examined the serum metabolome. We did so because gut microbial-derived metabolites must transit through the blood in order to reach the lungs and because effects of the microbiome on O_3_-induced AHR involve the gut rather than the lung microbiome [1]. However, though we think it unlikely, we cannot rule out the possibility that changes in the serum metabolome observed in antibiotic-treated versus control mice exposed to O_3_ were the result of effects of the antibiotics on the lung rather than the gut microbiome. On a technical note, we normalized our data by using equivalent volumes of serum from each mouse, similar to the method used by other investigators examining serum metabolomics [75, 76]. While this is a convenient and widely used method, we cannot rule out the possibility that changes in eating or drinking behavior related to O_3_ exposure or antibiotic treatment affected the blood volume and thus the concentration of metabolites or that clotting of blood during the generation of serum induced changes in some metabolites.

It is interesting to note that while antibiotics appropriately attenuated many known bacterial-derived metabolites, O_3_ had similar effects on many of the same metabolites (Fig 2). Since production of many of these metabolites requires reactions catalyzed in the liver in addition to reactions catalyzed within bacteria, it is conceivable that O_3_-induced changes in these metabolites reflect known effects of O_3_ on the liver [23, 53]. However, we cannot rule out the possibility that O_3_ exposure per se causes changes in the gut microbiome, perhaps as a result of changes in bile acids (Table 3) which have bacteriostatic effects.

In summary, we observed marked effects of O_3_ exposure on the serum metabolome that differed in control mice and mice in which the gut microbiome had been depleted with antibiotics. In particular, O_3_-induced changes in serum lipids were markedly different in control and antibiotic-treated mice. Microbial-dependent changes in some of these lipids, notably bile acids and long chain fatty acids, as well as changes in the polyamines, spermine and spermidine, may account for the role of the microbiome in pulmonary responses to O_3_, since each of these metabolites has the capacity to alter airway responsiveness and neutrophil recruitment.

## Supporting information

S1 Table Ozone- and antibiotic-induced changes in each of the metabolites identified. Sheet 1—Heatmap. The blue shaded areas indicate significant (p<0.05, q<0.10) effects on two-way ANOVA. Fold change results are the ratio of mean lipid metabolite scaled peak area in mice treated with regular drinking water or antibiotic-supplemented drinking water and exposed to air or ozone, as described in Table 2. Red and green shading indicates metabolites with significantly (p<0.05, q<0.10) increased (red) or decreased (green) values. p and q values for the various comparisons along with mean scaled peak areas for each group are shown in the columns on the right.
(XLSX)

S2 Table Ozone- and antibiotic-induced changes in each of the metabolites identified–Boxplots. A scaled intensity for each metabolite was calculated by dividing the raw area counts for each mouse by this median so that the median across all mice in all groups was now equal to 1, as described in Fig 2. This scaled intensity is presented on the y axis. For each group, the + indicates the mean value and the line in the center of bar indicates the median. The upper and lower limits of the bar are the upper and lower quartile and the top and bottom of the error bars are the maximum and minimum of the distribution. Extreme data points are indicated by symbols outside of the maximum and minimum of the distribution. Metabolites are organized alphabetically. n = 8 per group.
(XLSX)
